# Review of Chromatographic Bioanalytical Assays for the Quantitative Determination of Marine-Derived Drugs for Cancer Treatment

**DOI:** 10.3390/md16070246

**Published:** 2018-07-23

**Authors:** Lotte van Andel, Hilde Rosing, Jan HM Schellens, Jos H Beijnen

**Affiliations:** 1 Department of Pharmacy & Pharmacology, Antoni van Leeuwenhoek, The Netherlands Cancer Institute and MC Slotervaart, 1066 CX Amsterdam, The Netherlands; h.rosing@nki.nl (H.R.); j.beijnen@nki.nl (J.H.B.); 2 Division of Pharmacology, Antoni van Leeuwenhoek, The Netherlands Cancer Institute, 1066 CX Amsterdam, The Netherlands; j.schellens@nki.nl; 3 Department of Clinical Pharmacology, Division of Medical Oncology, The Netherlands Cancer Institute, 1066 CX Amsterdam, The Netherlands; 4 Department of Pharmaceutical Sciences, Faculty of Science, Division of Pharmacoepidemiology and Clinical Pharmacology, Utrecht University, 3584 CG Utrecht, The Netherlands

**Keywords:** marine-derived drugs, cancer, bioanalysis, chromatography

## Abstract

The discovery of marine-derived compounds for the treatment of cancer has seen a vast increase over the last few decades. Bioanalytical assays are pivotal for the quantification of drug levels in various matrices to construct pharmacokinetic profiles and to link drug concentrations to clinical outcomes. This review outlines the different analytical methods that have been described for marine-derived drugs in cancer treatment hitherto. It focuses on the major parts of the bioanalytical technology, including sample type, sample pre-treatment, separation, detection, and quantification.

## 1. Introduction

For years, researchers have roamed the seas and oceans in search of organisms possessing chemicals that could exhibit therapeutic effects. These include a wide variety of creatures, such as tunicates, mollusks, sponges, bacteria, seaweeds, chordates, mangroves, sea hares, and sharks [1,2,3]. Therapeutic indications include cancer, Alzheimer’s disease, thrombosis, hypertension, psoriasis, asthma, and pain [4,5,6,7]. Ruiz-Torres et al. (2017) have outlined the increase in manuscripts published about marine-derived compounds, and more specifically, the substantial increase in these compounds studied for cancer treatment over the last several years, with about 125 publications in 2016 [8]. This nicely demonstrates the increasing information and insights researchers have gathered with regards to marine-derived anticancer drugs.

Sea-based organisms have obtained the ability to produce chemicals, of which some might be cytotoxic, because they have learned to live under relatively bitter and hostile conditions in the marine environment. Biological evolution has therefore enabled them to acquire and to select these chemicals to survive [1] and to compensate for their lack of physical protection [9]. The extreme conditions involve high salt, high pressure, lack of oxygen and light, and extremely high or low temperatures [7,10]. Furthermore, these organisms suffer fierce survival competition, and the chemicals, or secondary metabolites, help marine organisms to survive, reproduce, and resist predators [7,10]. The therapeutic agents derived from these chemicals can be unaltered natural products, chemically altered derivatives, or their structure might have been inspired by natural marine products [11].

Unfortunately, the supply of organisms is not endless, and ecological constraints are often limiting research in this field. The challenges with regard to the limited availability of the compounds in nature are demonstrated by the narrative of trabectedin. Despite early proof that trabectedin seemed to be effective in cancer treatment, development delays were encountered due to its low natural abundance, and efforts had to be made to produce sufficient amounts on a large scale [9]. In fact, this limitation of the scaleup process has oftentimes been the reason for the discontinuation of clinical development [12]. Drug development is in itself a long journey but even more so if having to deal with ecological considerations when accessing the marine source of the compounds.

Many marine-derived compounds have complex molecular structures and therefore different challenges arise. Their structural elucidation and chemical synthesis might be difficult, and the complex molecules may include many potential bioactive moieties, which make it difficult to identify a single mechanism of action [13,14].

Despite these challenges, the discovery of new cancer treatments originating from marine-derived animals or plants is very much in the spotlight today. From the numerous discoveries of the new chemical entities done so far, some compounds have progressed into clinical trials. Clinical trials investigating safety and toxicity also require the characterization of the pharmacokinetic profile of these potential therapeutics. In order to accurately describe the pharmacokinetic profiles in biological fluids, such as plasma, whole blood, serum, and urine, (validated) bioanalytical methods are indispensable.

The first marine-derived compound approved by the United States (US) Food and Drug Administration (FDA) was cytarabine (Cytosar U^®^) in 1969, followed by trabectedin (Yondelis^®^), eribulin mesylate (Halaven^®^), brentuximab vedotin (Adcetris^®^), and midostaurin (Rydapt^®^) in the years 2007, 2010, 2011, 2013, and 2017, respectively.

Most marine-derived compounds possess a few defining characteristics. First of all, often very low doses are given to patients, and they are administered almost exclusively via intravenous infusion (i.v.). The compounds are typically large molecules (400–1700 Da), especially the depsipeptides, and they are often hydrophobic. These attributes make them fascinating compounds in terms of pharmacokinetics but also from a bioanalytical point of view. For instance, the hydrophobic drugs tend to distribute massively into peripheral tissues, which is demonstrated by high volumes of distribution. This means that plasma levels can be rather low at all times and that bioanalytical methods to quantify the drug in the plasma thus need to be highly sensitive.

### Focus of This Review

The demands for sensitive and specific analytical technologies for the determination and quantification of cytotoxic drugs in biological matrices are high. These methods provide the means to construct pharmacokinetic profiles, to determine drug exposure during treatment, and to evaluate if target concentrations are reached. Moreover, they can be used to quantify drugs and their metabolites in excreta to investigate excretion profiles and metabolism. This information is pivotal in drug development and clinical application but also required by regulatory agencies, such as the FDA and the European Medicines Agency (EMA). This review outlines the available methods for analyses of marine-derived anticancer agents in biological matrices. Furthermore, information concerning the sensitivity capabilities of each method is provided. This review provides a comprehensive list of analyses of marine-derived compounds in various biological matrices published over the last few decades, which could potentially also serve as a guide for newly discovered or synthesized drugs from marine origin.

## 2. Marine-Derived Anticancer Drugs

A huge number of marine-derived compounds are described in over 700 pages in the book titled “Handbook of Anticancer Drugs from Marine Origin” (2015), which far exceeds the practicality of this review. Instead, successful anticancer compounds, those that have progressed into clinical development, described in three relatively recent reviews by Mudit and El Sayed (2016), Newman and Cragg (2016), and Palanisamy et al. (2017) were used as basis of this review and cover the relevant anticancer compounds of current interest [2,15,16]. 

Bioanalysis refers to the analysis of compounds, particularly drugs, in biological matrices and encompasses therefore a large range of applications. Manuscripts were included in this review if they described some type of chromatography for bioanalysis, because this is the most widely applied and available method for drug analysis. Also, compounds that have progressed into clinical trials but failed were included in our literature search, because knowledge about the analysis of these agents might be valuable, especially as it is not uncommon to chemically synthesize derivatives of the potentially failed drugs. Manuscripts published before 1990 were excluded from the review. Publications were also excluded if the method described was not applied to biological samples.

Cytarabine, although being the first identified anticancer drug of marine origin, is excluded from the review. Cytarabine is a nucleoside analogue that is structurally very different but also much simpler than the other marine-derived agents. The bioanalysis of this group of compounds has been extensively reviewed elsewhere [17]. Moreover, the antibody-drug conjugates (ADCs) with payloads (monomethyl auristatin E [MMAE]) derived from the marine-derived cytotoxic agent auristatin were excluded as well, because bioanalysis of this class of drugs is a subject of its own. Drugs belonging to this group are ASG-15ME, ASG-67E, brentuximab vedotin, depatuxizumab mafodotin, denintuzumab mafodotin, enfortumab vedotin, glembatumumab vedotin, GSK2857916, indusatumab vedotin, ladiratuzumab vedotin, lifastuzumab vedotin, pinatuzumab vedotin, polatuzumab vedotin, tisotumab vedotin, and vandortuzumab vedotin.

Within the PubMed database, keyword searches for ‘drug name AND bioanalysis’, ‘drug name AND quantification’, ‘drug name AND assay’, and ‘drug name AND determination’, where ‘drug name’ included all synonyms, both generic and brand name, were applied. The careful analysis of titles and abstracts led to a selection of manuscripts describing chromatographic bioanalytical methods to determine marine-derived anticancer drug concentrations in biological matrices. 

## 3. Bioanalysis of Marine-Derived Anticancer Drugs

### 3.1. Drugs

In total, 35 marine-derived anticancer drugs are included in the three aforementioned reviews [2,15,16]. Table 1 lists the marine-derived drugs approved for cancer treatment or still under investigation in clinical trials. It includes 20 compounds, of which 5 are approved, 6 are still being investigated in phase I clinical trials at escalating doses, 6 are currently in phase II clinical trials, and 3 have entered phase III clinical trials. Some drugs mentioned in the reviews by Mudit and El Sayed [2], Newman and Cragg [15], and Palanisamy et al. [16] have been discontinued from clinical trials or no active clinical trials can be found in the ClinicalTrials.gov database. These compounds can be found in Table 2, and the corresponding bioanalytical methods were still included in the present review whenever available. Seven of these drugs are natural products (didemnin B, marizomib, plitidepsin, PM060184, pipecolidepsin A, stellatolide A, and trabectedin); all others are derivatives or analogues thereof.

Of those that are still under investigation or approved for cancer treatment, all but one are administered via i.v. Midostaurin, approved for acute myeloid leukemia in May 2017, is an oral drug given at a dose of 50 mg twice daily, the highest dosage compared to the other selected compounds in Table 1. This could be related to limited oral bioavailability, but unfortunately this remains unknown [30]. Plinabulin is given at 30 mg/m^2^, and all other drugs are administered at dosages between 0.5–5 mg/m^2^. Most compounds distribute widely to peripheral tissues, with volumes of distribution at steady state (V_ss_) ranging from approximately 18–5000 L [19,24,30,34,46,47,48,49,50,51,52,53,54,55,56,57,58]. UCN-01 and CEP-2536 are the exceptions, with lower V_ss_ ranging from 4 to 14.3 L and 0.35 to 44 L, respectively [59,60,61]. 

Table 3 includes all manuscripts covering bioanalytical methods developed to quantify marine-derived anticancer therapeutics in biological matrices. In total, 23 manuscripts describing bioanalysis were included. Compounds that were included in the above three reviews but for which no bioanalytical methods were found include becatecarin, CEP-2563, didemnin B, edotecarin, enzastaurin, lestaurtinib, marizomib, pipecolidepsin A, stellatolide A, plinabulin, and PM060184.

**Figure 1:**
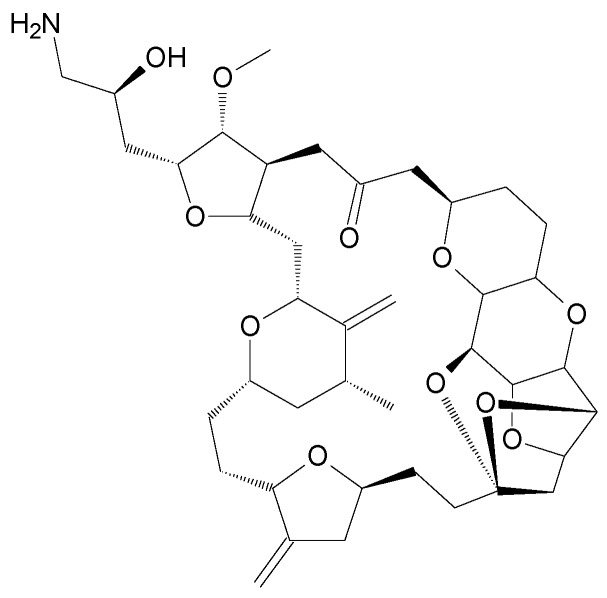
Chemical structure of the macrolide eribulin [64].

**Figure 2:**
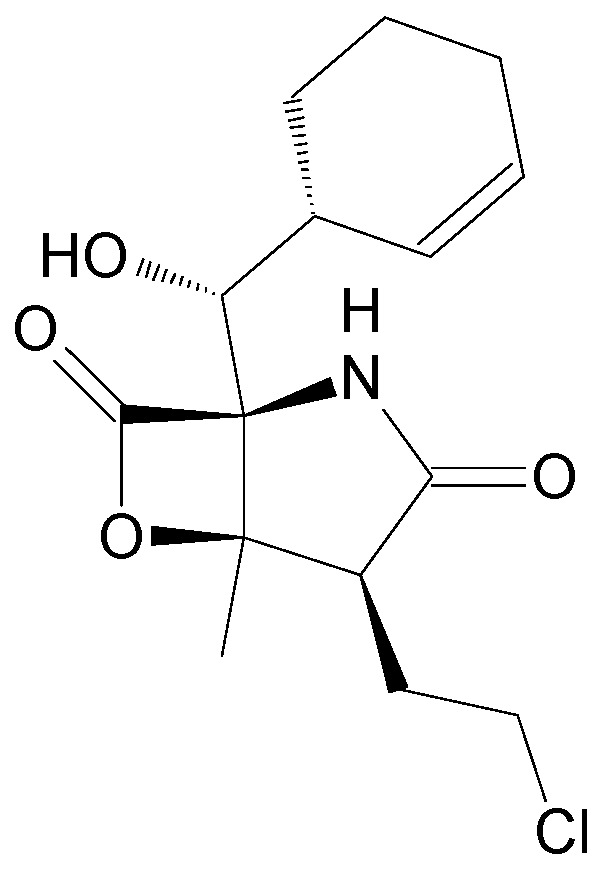
Chemical structure of marizomib, a salinosporamide [39].

**Figure 3:**
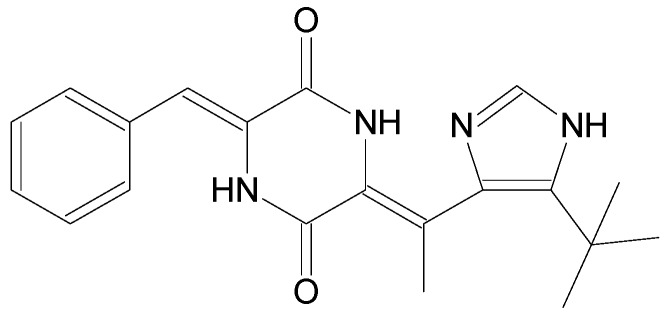
Chemical structure of the piperazine derivative plinabulin [86].

**Figure 4:**
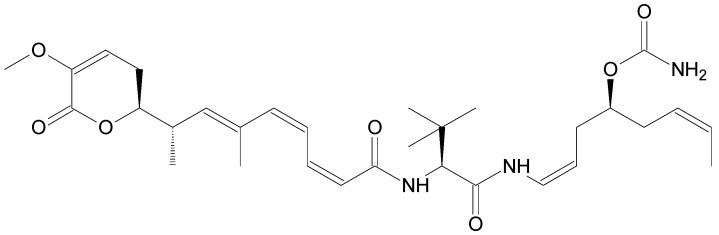
Chemical structure of PM060184, a polyketide [39].

**Figure 5:**
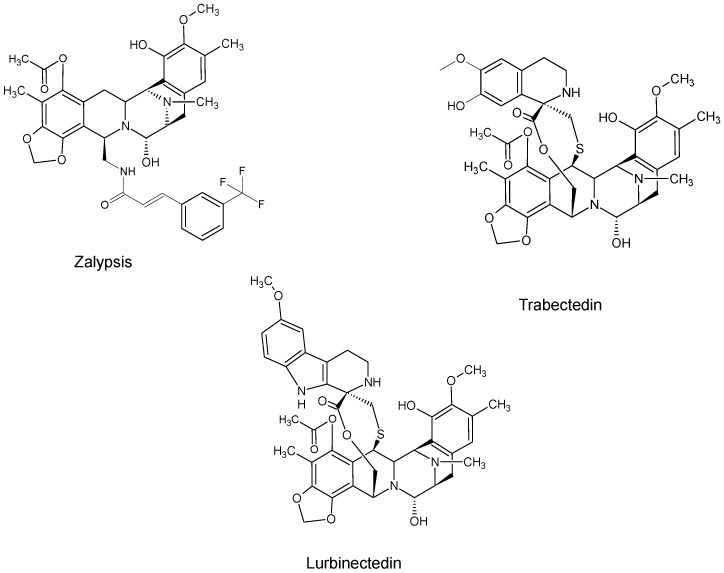
Chemical structures of the isoquinolines zalypsis [39], trabectedin [90], and lurbinectedin [65].

**Figure 6:**
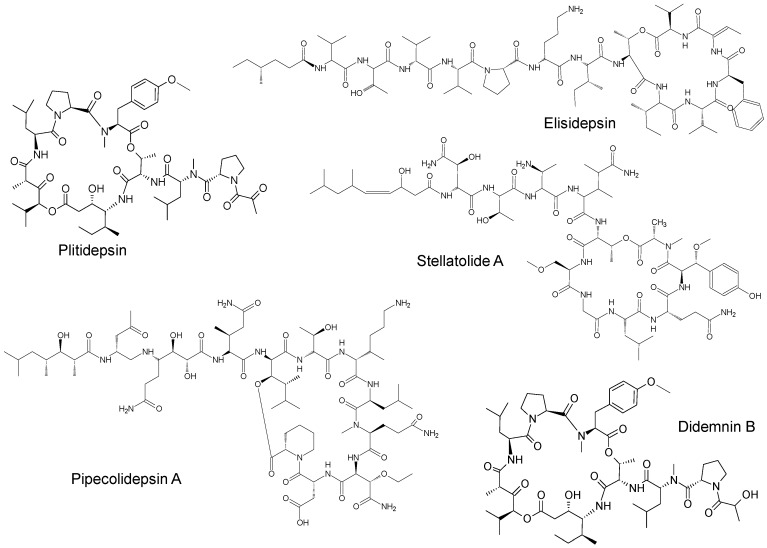
Chemical structures of the depsipeptides didemnin B, elisidepsin [15], pipecolidepsin A [13], plitidepsin [75], and stellatolide A [15].

**Figure 7:**
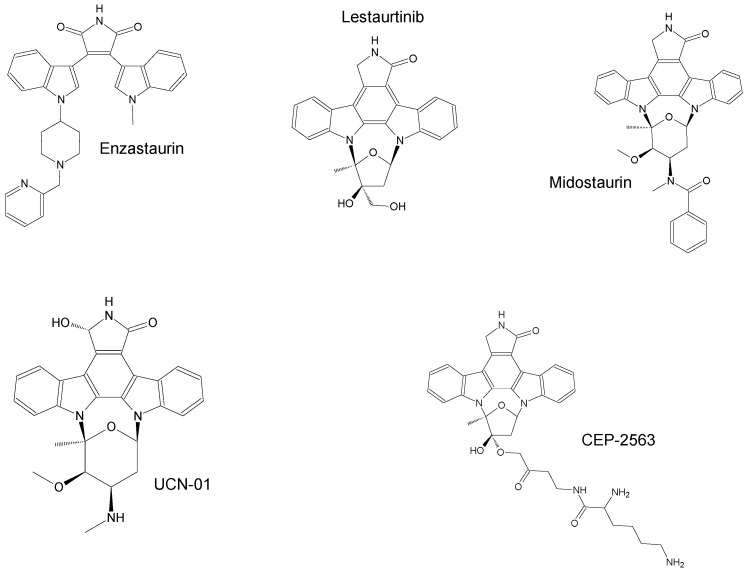
Chemical structures of the staurosporine derivatives CEP-2563 [16], enzastaurin, lestaurtinib, midostaurin, and UCN-01 [91].

**Figure 8:**
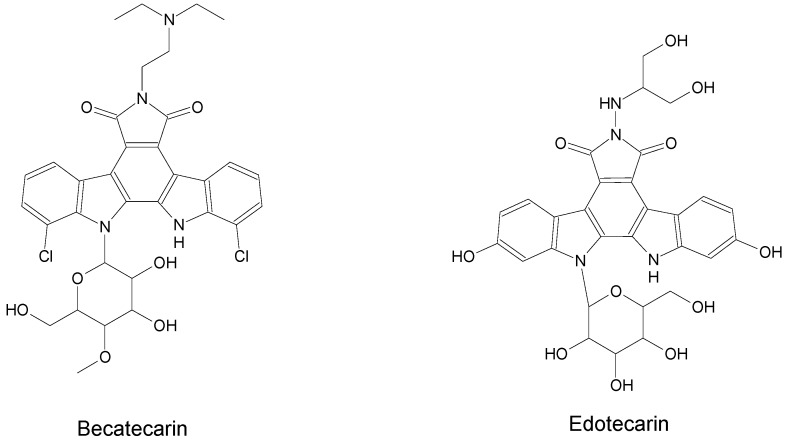
Chemical structures of the rebeccamycin derivatives becatecarin and edotecarin [92].

The compounds included in the present review can be grouped together according to their chemical structures. Eribulin belongs to the macrolides [15] (Figure 1), marizomib is a salinosporamide [85] (Figure 2), plinabulin is a piperazine derivative [86] (Figure 3), and PM060184 is a polyketide [15] (Figure 4). The isoquinoline alkaloids include zalypsis, trabectedin, and its structural analogue lurbinectedin [15] (Figure 5). The largest groups are the depsipeptides (didemnin B, elisidepsin, pipecolidepsin A, plitidepsin, and stellatolide A) [15] (Figure 6) and the indolocarbazoles (CEP-2563, enzastaurin, lestaurtinib, midostaurin and UCN-01), all derivatives of staurosporine [87,88], (Figure 7) and becatecarin and edotecarin, derivatives of rebeccamycin [89] (Figure 8).

**Figure 9:**
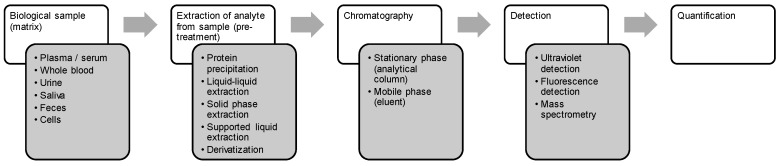
General set up of drug analysis.

The chromatographic bioanalytical methods can be divided in different steps (Figure 9), starting with the collection of a biological sample. Next, the drug, or analyte, needs to be extracted from the biological matrix, and the sample is cleaned up to such an extent that all other (endogenous) compounds extracted from the sample do not interfere with analysis. Thereafter, the sample is injected onto a chromatographic system, and the mobile phase together with the stationary phase enable the separation of the analyte of interest from the other components in the sample. Finally, the analyte is detected by a detector. Derivatization might be necessary prior to sample cleanup if an ultraviolet (UV) detector or a fluorescence detector (FLD) is used. The next paragraphs describe these individual steps of the drug analysis. 

### 3.2. Matrices

#### 3.2.1. Plasma and Serum

Most bioanalytical assays have been developed for the plasma and serum matrix. Of the 23 manuscripts included, 21 cover concentration determinations in plasma or serum. Generally, this is done to assess plasma pharmacokinetic characteristics and to link this to clinical outcomes (antitumor efficacy and/or toxicities). Ideally, quantification of the drug is done at its site of action, but this is (usually) not possible. Instead, blood samples are taken, plasma or serum is obtained by centrifugation, and the drug concentrations are taken as a substitute for the concentrations at the site of action. It is relatively simple to draw a blood sample, and although invasive, the burden of multiple venipunctures can be reduced by placing a peripheral intravenous catheter. This way, a complete pharmacokinetic profile could technically be constructed with a single vein puncture. 

Because the only difference between plasma and serum is the presence of coagulation factors, the concentrations in plasma and serum are usually regarded as equivalent. Serum samples are, however, cleaner compared to plasma because of the removal of clotting factors. This could be beneficial, because coagulants can contaminate the analysis instrumentation (i.e., the analytical column). The downside is that it takes some time for the blood to coagulate, so sample processing is relatively more tedious compared to obtaining plasma. On the other hand, a serum sample is slightly more concentrated than a plasma sample, because the coagulation factors have been removed from the sample. No bioanalytical methods were found for lestaurtinib and enzastaurin, but pharmacokinetic studies have been published describing serum pharmacokinetics of these compounds [93,94,95], indicating that assays exist but are not in the public domain as far as we know. 

#### 3.2.2. Whole Blood

Whole blood samples are less easy to process than plasma samples due to their viscosity and need to be handled more carefully than other sample types. For instance, to homogenize and thus distribute compounds evenly, a whole blood sample should only be shaken very gently and not too vigorously. An advantage of whole blood can be the presence of hemoglobin in the blood, which might have a stabilizing effect, making the analyte present less prone to photo-oxidation compared with plasma [96]. However, hemoglobin could also interfere with analysis. Whole blood analyses are considered when a drug is bound to red blood cells, such as plitidepsin [73,74,75]. Plitidepsin concentrations in whole blood are approximately fourfold higher than its plasma levels, so red blood cells are an important distribution compartment in this case [97,98]. Quantifying the drug in red blood cells, therefore, has added value. A bioanalytical method has been developed to quantify eribulin mesylate in whole blood as well, but there were no significant differences in the plasma and whole blood concentrations measured [64,99]. 

#### 3.2.3. Urine

Drug concentrations are frequently measured in urine to get insight into drug and metabolite excretory pathways. Urine collection is fairly easy: it is non-invasive, and urine quantities are large. Drug levels can often be measured for longer periods of time after administration compared with blood [100]. It also adds information in terms of pharmacokinetics. By dividing the amount of drug excreted unchanged by the dose given, the fraction of the amount entering the general circulation that is excreted unchanged (f_e_) can be calculated. This provides a quantitative measure of the contribution of renal excretion to overall drug elimination [101]. The drawbacks of urine analysis, however, include the large variety in urine composition between patients but also between intervals/timepoints. Furthermore, in order to obtain complete excretion data, urine collection needs to be complete, and this is even more challenging in a clinical setting when the patients need to collect samples at home. Another potential problem with urine analysis is non-selective adsorption of the analyte to the container wall. Urine is an aqueous fluid, which means that hydrophobic analytes might prefer to adhere to the container wall than to be in the aqueous solution. This specific type of nonselective adsorption has been described for plitidepsin only [75], despite the fact that this problem would seem to be more prevalent due to the hydrophobic nature of most of the compounds discussed here. A reason for this is that not much urine analysis has been executed in the first place. Eribulin is less hydrophobic than, for instance, plitidepsin, and for the UCN-01 assay, 0.5% polyoxyethylene (20) sorbitan monolaurate was added to the tubes to prevent adsorption from taking place. Beumer et al. also briefly mentioned the prevention of the adsorption of trabectedin to the container wall by adding bovine serum albumin to the tubes before urine collection [90]. Mostly, urine drug analysis is done to support the monitoring of renal excretion of the drug in (pre-)clinical studies [70,73,102] and more specifically to support human mass balance studies, in which excretory pathways are investigated [64,75,98,99,103].

#### 3.2.4. Saliva

It is not uncommon to measure drug concentrations in saliva. It might be preferred over blood sampling, because it is non-invasive and collection is fairly easy, causing much less stress and discomfort [104]. Saliva analysis could, in fact, even be used for therapeutic drug monitoring (TDM); however, if concentrations are linked to plasma concentrations, then the risk for overestimation owing to preferential partitioning into the saliva is high [105], and sample volumes are usually small [100]. Moreover, oral contamination might occur and intra- and inter-patient variability in pH can impact quantification [104]. The only publication included here that quantifies drug levels in saliva is for UCN-01 analysis. In this case, it was believed that saliva concentrations reflected free plasma concentrations, and this unbound fraction is responsible for the pharmacological effect [82,100,106]. Thus, the method was developed not only for plasma but for saliva as well. Dees et al. investigated the relationship between saliva concentrations of UCN-01 and hypotension. Hypotension was the most severe adverse event that occurred during the clinical trial with the drug [59]. 

#### 3.2.5. Feces

Quantification of drugs in feces could be considered for bioanalysis as part of a mass balance study where excretory pathways of a drug and its metabolites are investigated. However, the major concern is that feces composition varies greatly, and this may have an effect on the sample preparation recovery [107]. One manuscript included in the review demonstrates drug quantification in feces. The method was validated to support a human mass balance clinical trial in which the excretion of eribulin was investigated and hence included quantification in feces [64]. The mass balance study had shown that the majority of eribulin and eribulin-metabolites was excreted via the feces [99], meaning that the ability to quantify eribulin mesylate levels reliably had substantial added value. Potential problems were overcome by diluting the feces in water, in order to obtain samples with similar compositions [99]. 

#### 3.2.6. Cells

Occasionally, drug concentrations are measured inside tissues or cells. The obvious advantage here is that drug concentrations are measured directly in the tumor and that drug distribution in the tumor can be determined, whereas plasma measurements are usually a surrogate for drug determination at the site of action. The drawbacks are that cells need to be isolated, counted and lysed, and tissues need to be homogenized even before sample pre-treatment. One publication describes the quantification of a drug in cells. In this case, it determined trabectedin concentrations in the liver cells and tumor cells originating from xenograft mice to investigate the tissue distribution of trabectedin [80]. The authors believed that the available data on plasma concentrations did not predict accurately the drug levels achievable in tumor or tissues, hence the reason for the development of this method. 

### 3.3. Sample Preparation and Recovery

Before analysis, samples are usually pre-treated in order to extract the analyte from the matrix abundant with endogenous, interfering compounds. Moreover, because marine-derived drugs are administered at low doses, the analyte most likely needs to be concentrated as well. Sample preparation can be highly specific to extract one analyte of interest. However, when multi-drug assays are to be developed, the sample cleanup should not be too specific, otherwise some of the analytes of interest might be lost. Below is an overview of the sample pre-treatment methods applied to extract marine-derived drugs from biological samples.

#### 3.3.1. Protein Precipitation

The sample pre-treatment method generally employed most often is protein precipitation (PP). Plasma and serum especially contain many proteins, which should be removed from the sample before analysis, because they can interfere with or contaminate analysis, also clogging the analytical column [108]. Precipitation of proteins is usually done with organic solvents, such as acetonitrile or methanol (or a mixture of the two), and often contains an acid, such as formic acid (FA) or trichloroacetic acid (TCA). In the included methods, the recovery of the analyte after PP is consistently high, with recoveries over 80% [69,78,79,82,83]. Recoveries above 100% have been reported and could occur for two reasons. First of all, it could be due to ion enhancement in the source of the mass spectrometer (MS) [64]. Secondly, it could be due to adsorption of the analyte to the equipment/materials in a sample free of matrix [78].

#### 3.3.2. Liquid–Liquid Extraction 

Liquid–liquid extraction (LLE) can occur when two immiscible solvents are used, resulting in two phases, an organic and an aqueous phase. The separation of the analyte from other (endogenous) compounds in the sample is based on the different distributions of these compounds in the two phases. The advantages of LLE include the low cost and easy application. A downside, however, is the fact that large volumes of organic solvents are often used. LLE is usually performed with ether (diisopropyl ether, *tert*-butyl methyl ether, or diethyl ether), ethyl acetate, chloroform, and *n*-butanol, possibly with pH modifications, and is therefore suitable for extraction of lipophilic compounds [109]. The next step is to evaporate the organic phase, because these solvents are not compatible with LC-MS/MS, at least when large injection volumes are used (this is generally not a problem if only 1 μL is injected onto the analytical column). This method has been applied for the lipophilic drugs elisidepsin, eribulin, midostaurin, plitidepsin, and zalypsis. In these cases, the analyte is removed from the aqueous plasma layer and partitioned into the organic layer. Whenever reported, the sample pre-treatment recovery was moderate to high (57–115%) with three exceptions: LLE with a mixture of ethyl acetate, methanol, and ethanol resulted in an extraction recovery of 33–45.3% for eribulin, whereas this was more than 60% for another method published using the same extraction solvent. The authors claim this to be due to matrix effects [63].

#### 3.3.3. Solid Phase Extraction 

Solid phase extraction (SPE) remains a popular cleanup method as well. The advantage is that it often results in very clean samples, because the SPE columns are very efficient at retaining endogenous, interfering substances. Despite this major advantage, SPE remains relatively laborious and costly, especially compared with PP and LLE. During SPE, the analyte is retained on a sorbent, after which matrix interferences are removed by a washing step. Then the analyte is eluted from the SPE column using an elution solvent. This sample pre-treatment method has been applied in four cases included in this review [71,74,76,77]. C18 cartridges and cyano cartridges were used for plitidepsin and trabectedin, respectively. Cleanup of samples containing trabectedin using SPE resulted in high recoveries (>87%) [76,77]. It resulted in lower recoveries for plitidepsin (>55%) [71,74]. However, it was found to be reproducible.

#### 3.3.4. Supported Liquid Extraction 

Supported liquid extraction (SLE) is a relatively new technique but analogous to LLE. The difference between the two is that the two immiscible solvents are not mixed together, but instead, they are immobilized on an inert support, and the organic phase is used to extract the analyte from the SLE column [110]. It has been used only for the sample cleanup of lurbinectedin [65]. Despite its presumed ability to have thorough cleanup of the samples, getting rid of most endogenous compounds, extraction recovery was rather low and very variable (data on file). In this case, a stable isotopically labeled (SIL) internal standard is recommended to ensure that the variable recoveries are sufficiently corrected for. Though, the signal-to-noise ratio was quite high, with values around 7 for the lower limit of quantification (LLOQ), meaning that SLE was able to thoroughly clean up these biological samples. 

It is well known that phospholipids, proteins that are highly abundant in plasma, cause major ion suppression effects in mass spectrometric detection and thereby influence analysis [111]. LLE and the analogous SLE are efficient in extracting analytes from phospholipids, resulting in clean samples. During SPE, on the other hand, efficient phospholipid removal is not a guarantee. Instead, specific types of cartridges need to be used in order to separate the analyte from the phospholipids. These include, for instance, strong cation exchange sorbents or hybrid SPE [111]. 

#### 3.3.5. Derivatization 

Some sample pre-treatment methods involve a derivatization step in order to enable ultraviolet (UV) detection or fluorescence detection. The obvious drawback is that this extra step makes the method more laborious and complicated. Two methods have been described to quantify plitidepsin levels in biological fluids, by first derivatizing plitidepsin using trans-4-hydrazino-2-stilbazole to enable fluorescence detection [71,74]. With the advent of liquid chromatography coupled with mass spectrometry detectors, the use of this type of analysis has become less prevalent. 

### 3.4. Analytical Methods

#### 3.4.1. Chromatography

As it was a criterion for inclusion, all methods make use of high performance liquid chromatography (HPLC). During HPLC analysis, a sample is brought onto an analytical column, the stationary phase, and eluted from it using a suitable eluent, the mobile phase. 

All manuscripts included in this review describe the use of reversed-phase (RP) liquid chromatography (LC). On RP columns, polar compounds elute before non-polar compounds. The most widespread used RP columns have regular C18 stationary phases, most notably Polaris C18, μBondapak RP-18, Hypersil-5 ODS, and the Symmetry C18 columns. A few assays report the use of other types of RP columns. For plitidepsin analysis, a Zorbax Bonus-RP column has been applied, which has C14 instead of C18 alkyl chains, with an amide linkage in between [72]. For lurbinectedin, an ACE pentafluorophenyl (PFP) column has been reported, which has a PFP group attached to the C18 alkyl chain [65]. RP analytical columns with phenyl chemistries have been utilized for the analysis of UCN-01 [82,83]. Unfortunately, justification has not always been given for those choices. 

Because most compounds included in the review are hydrophobic, they elute from the analytical column with relatively high concentrations of organic mobile phases. The most widespread mobile phases used include water as the aqueous phase and acetonitrile or methanol as the organic phase. Modifiers include ammonium acetate, ammonium formate, formic acid, triethylamine, tetrahydrofuran, trifluoroacetic acid, and phosphoric acid. A few authors justify their choice of mobile phase. 

For instance, Celli et al. have explained that the use of formic acid as the modifier has the advantage that the formation of sodium and potassium adducts of plitidepsin are reduced, and higher MS sensitivity is thereby obtained [70]. Moreover, trifluoroacetic acid could inhibit the ionization of the analyte and should therefore not be added to the mobile phase. The addition of ammonium acetate or ammonium formate would produce ammonium adducts and lower the sensitivity [70,73]. However, our group has reported that ammonium acetate added to the mobile phase could significantly improve peak shape in the plitidepsin assay [75]. Despite other groups reporting the disadvantage of the ammonium adduct formation for sensitive MS detection, this was not a problem in the latest assay, because sensitivity was more than sufficient [75]. 

Rosing et al. have also experimented with various modifiers in the assays for trabectedin quantification [77]. They found that ammonium acetate and acetic acid reduced MS electrospray sensitivities greatly. However, small amounts (5 mM) of ammonium acetate were added, because buffer capacity was needed to separate trabectedin from its internal standard. On the other hand, formic acid needed to be added as well, because this greatly improved sensitivity [77]. 

An isocratic system, where the mobile phase composition does not change throughout the analytical run, has been reported for midostaurin, plitidepsin, trabectedin, and UCN-01, for instance, by van Gijn et al. [66] and Illmer et al. [68] for the quantification of midostaurin. The first group quantified midostaurin and three potential metabolites [66]. When the method was adapted to include a fourth potential metabolite, isocratic elution seemed to be inappropriate [67]. Although isocratic elution should in theory lead to enhanced separation of analytes, gradient elution was applied, and it showed to be successful in separating the parent drug and all four metabolites. Moreover, triethylamine had a positive effect on the chromatography overall [67]. Kurata et al. stated that the addition of triethylamine to the isocratic mobile phase reduced peak tailing of UCN-01 [81]. 

It is surprising that none of the publications describe ultra-high-performance liquid chromatography (UHPLC). This type of chromatography has been available since 2004, and it has improved sensitivity, selectivity and speed owing to the smaller particle size (2 μm) [112]. 

#### 3.4.2. Detection

##### UV Detection

UV detection is not commonly used for the analysis of marine-derived compounds; in only two reports is analyte detection by UV described [76,82]. The absorbance wavelengths for trabectedin and UCN-01 are 210 nm and 295 nm, respectively [77,82]. The lowest LLOQ achieved using UV detection was 1 ng/mL for trabectedin with sample volumes of 500 μL [76]. 

##### Fluorescence Detection

Fluorescence detection has been described for the detection of midostaurin, plitidepsin, and UCN-01 [66,67,68,71,74,81,82,83]. A compound needs to possess a chromophore to enable fluorescence detection, hence some analytes lacking this need to be derivatized before enabling the detection by fluorescence [71,74]. The lowest LLOQ achieved using fluorescence detection was 0.2 ng/mL with sample volumes of 50 μL (UCN-01) and 100 μL (midostaurin) [67,81].

##### Mass Spectrometry 

Most recent articles make use of mass spectrometry (MS) detection, often consisting of a triple quadrupole detector after electrospray ionization (ESI) or atmospheric pressure chemical ionization (APCI). The reason for this shift is that MS is in general more sensitive than UV, and fluorescence detection has shown improved selectivity, and it has become more readily available. The marine-derived drugs are commonly administered at very low doses, meaning that lower limits of quantification (LLOQs) in the nanograms and even pictograms per milliliter range are often required. This shift towards MS is seen for a number of compounds included in this review. 

For instance, Rosing et al. have developed a method to quantify trabectedin in a linear range of 1–50 ng/mL [76]. An HPLC-UV method was validated first. However, after the first clinical trials in humans, the assay seemed to be lacking in sensitivity, hence a more sensitive HPLC-MS/MS method was developed [77]. A similar trend was seen for midostaurin [66,67,68,69]. The recent method for midostaurin quantification was developed for high-throughput analysis and, more specifically, for TDM-purposes [69]. Interestingly, no metabolites were taken into account for this assay, whereas they were included in the older assays. Some of these metabolites have been shown to be active, so it might have been appropriate to include the metabolites in the assay meant for TDM [113]. 

As just mentioned, MS provides superior sensitivity, hence it should be used in case of possible sensitivity problems. Sensitivity issues can arise for numerous reasons. For instance, Rosing et al. have emphasized the challenges in terms of sensitivity that occurs for those drugs that are administered at prolonged infusion schedules [77]. Zalypsis, elisidepsin, plitidepsin, and trabectedin have all been administered for over 24 h [50,58,114,115], and UCN-01 has even been infused to cancer patients for over 72 h [116]. This fact, together with the low dose and high volumes of distribution require the need for sensitive methods. Moreover, sensitivity issues have been described for plitidepsin besides the lengthy infusion times. Plitidepsin exists in two conformers (*cis* and *trans*), and the conformation equilibrium reaction is slow enough at room temperature for the two conformers to be separated as two chromatographic peaks. This is a well-known phenomenon for proline-containing peptides. In the case of plitidepsin, it is not desirable to distinguish between the two conformers, because they do not differ in clinical activity [117]. Moreover, lack of sensitivity can occur, because the MS signal is distributed over two chromatographic peaks, thereby reducing the sensitivity of the assay. This means that accurate quantification is especially challenging at low concentrations, and the assay needs to be sufficiently sensitive because of the low dose administered, long infusion times, and high volumes of distribution.

Interestingly, for the isoquinolines, the most pronounced ion observed in the mass spectrum corresponds to a loss of water from the molecule, a phenomenon that has not been described for any of the other compounds included in the review [65,77,78,79,80,84]. Allowing quantification based on the parent mass could therefore induce sensitivity problems as well. 

The lowest LLOQ achieved using MS detection was 0.01 ng/mL with sample volumes of 200 μL (zalypsis) and 500 μL (trabectedin) [77,84], whereas the lowest LLOQ achieved by UV detection was 1 ng/mL trabectedin with a sample volume of 500 μL [76].

Still, there are a few disadvantages. First of all, MS is costlier than UV and still not available in every laboratory, and secondly, matrix effects could seriously hamper analysis. Correct quantification is based on the ionization of the analyte. If co-eluting endogenous compounds in the matrix interferes with ionization, thereby causing ion suppression or ion enhancement, accurate quantification cannot be guaranteed [118].

### 3.5. Quantification

#### 3.5.1. Analytical Range

Assay sensitivity is very important, especially for those drugs that are administered at low doses and show large volumes of distribution. The analytical range should be appropriate for the therapeutic window or target concentrations to be reached, but the range should not be too broad either, because this could lead to non-linearity, which could impact the accuracy of the method. Ideally, at least 90% of the study samples should fall within the analytical range. Re-analysis is not preferred, especially if dilution integrity has not been tested for during method validation. Furthermore, samples with concentrations above the upper limit of quantification (ULOQ) can lead to carry-over problems, meaning that concentrations in the subsequent samples might be overestimated. Occasionally, adjustments to methods are necessary to accommodate for samples in clinical trials. For instance, an assay was developed to quantify trabectedin in human plasma. In a phase I trial with prolonged infusion of the drug, it appeared that the measured concentrations were lower than the anticipated analytical range for which the method was validated [76]. Hence, a new, more sensitive method was developed [77]. The analytical range was thus decreased from 1–50 ng/mL to 0.01–0.25 ng/mL. 

The importance of the analytical range is also demonstrated for elisidepsin, eribulin mesylate, and UCN-01. An assay to quantify elisidepsin had a range of 0.05–100 ng/mL [62]. A phase I clinical trial published in 2016 reported C_max_ values of ~100 ng/mL, and samples could be measured up to 336 h, covering the complete pharmacokinetic profile [119]. The published method is therefore suitable for the analysis of samples obtained after dosing patients as reported in the phase I clinical study. Perhaps a few samples could be above the ULOQ and would have to be diluted prior to analysis. Dilution integrity was, however, not tested during validation [62]. 

Eribulin mesylate methods have been developed and validated for plasma, whole blood, urine, and feces, with analytical ranges of 0.2–100 ng/mL (plasma), 0.5–100 ng/mL (whole blood and urine), and 0.1–25 μg/mL (feces homogenates) [64]. The drug has been registered at a dosage of 1.4 mg/m^2^ (equals 1.23 eribulin freebase), and the most recent pharmacokinetic data published report plasma C_max_ values ranging from 109 ng/mL (normal liver function) to 236 ng/mL (impaired liver function), meaning that not all samples could be measured by the published methods without the need for dilution [120]. The methods published did test for dilution integrity and appear appropriate [63,64]. 

An assay was developed for quantification of UCN-01 with an ULOQ of 100 ng/mL. The consequence was that samples collected in clinical trials [121,122,123] needed to be diluted 300 times prior to injection to ensure that concentrations fell within the calibration range [81,82]. A method published later had extended the analytical range up to 20 μg/mL, which means that even C_max_ samples from the phase I trials could be measured without dilution [82]. 

#### 3.5.2. Internal Standard

It is customary to use an internal standard to correct for variability in the sample pre-treatment, as well as sample analysis and detection (including matrix effects, such as ion suppression or enhancement when MS detection is used), thereby improving accuracy and precision of quantitation [124,125]. Preferably, an internal standard has similar physiochemical properties as the analyte of interest. Typically, two types are commonly used: structural analogues, both for UV and MS detection, and stable isotopically labeled (SIL) internal standards for MS methods; 21 out of 23 manuscripts report the use of an internal standard, of which only eight MS methods out of 14 make use of a SIL internal standard. It is highly recommended to use SIL internal standards for bioanalytical methods to compensate for matrix effects. Occasionally, the internal standard is a compound that is not even remotely similar to the analyte, which was for instance the case when propyl-*p*-hydroxybenzoate was used as the internal standard in an assay to quantify trabectedin in plasma and *N*-phenyl-1-naphthylamine in a midostaurin assay [68,76]. Availability and costs are probably the most determining factors in choosing the internal standard. Nevertheless, it seems as if the internal standards used in these assays fulfilled their purpose, with excellent accuracy and precision values reported. Two publications do not report the use of an internal standard at all [71,74]. In the first, it was mentioned that an appropriate internal standard was not available, but nevertheless, the accuracy and precision of the assay was acceptable. Interferences were investigated, and it was concluded that there were none that could influence quantification of plitidepsin. Interestingly, peak height was used for the quantification of plitidepsin in the second method, whereas the peak area was used in the first [71,74].

The first study discussed in this review to use a SIL internal standard was in 2004 for the quantification of trabectedin in plasma [78]. Our group has investigated whether the use of a SIL internal standard was always superior over other types of internal standards [124]. We have looked into various assays that were modified in order to substitute the structural analogue with a SIL internal standard, and we have demonstrated this for a marine-derived anticancer compound kahalalide F as well [126]. We found that a structural analogue often leads to insufficient accuracy and precision and concluded that a SIL internal standard usually corrected best for variation compared with a structural analogue, because its structure is more similar and hence the compound behaves more similarly in terms of recovery, ionization, and stability. The latter issue was also demonstrated for the kahalalide F assay: putative stability of the analyte was significantly prolonged using the SIL internal standard [126]. 

When a SIL internal standard is used, generally ^13^C or ^15^N isotopes are preferred over ^2^H-labeled internal standards, because these isotopes are more stable. Moreover, it has been shown that ^2^H-labeled internal standards do not always co-elute with the analyte of interest. This is due to slight differences in physiochemical properties, because ^2^H has stronger binding capacities than does hydrogen [124]. Co-elution is important, because it ensures similar matrix effects for internal standard and analyte and hence improved correction for variation [124]. Furthermore, differences in physiochemical properties can induce differences in extraction recovery due to exchange of ^2^H atoms with hydrogen atoms.

If, still a structural analogue is used, it should not resemble or correspond to any metabolites that could have been formed in vivo [124]. For instance, Rosing et al. (1998) used ET-729, demethylated trabectedin, as an internal standard, but a later mass balance and metabolite profiling study has detected this compound in feces [127]. It could not be confirmed if the product was indeed a metabolite or a degradation product, but regardless, its use as an internal standard is generally undesirable. This was addressed, and it was concluded that no ET-729 was present in patient samples after trabectedin administration [77]. The internal standard used in bioanalytical methods to quantify eribulin also used a structural analogue, where the NH_2_-group had been replaced by an OH group [63,64]. Technically, this could also result from in vivo biotransformations, but a mass balance study has revealed that it is not formed, hence the use of this internal standard was most likely appropriate [99]. The obvious advantage of using structural analogues is that they are often readily available and therefore much cheaper than a SIL internal standard, which requires dedicated synthesis.

Finally, it is worth mentioning that the importance of the internal standard’s resemblance to the analyte depends on the sample pre-treatment as well. A simple dilution or PP method might require less resemblance of the internal standard to the analyte than a liquid–liquid extraction (LLE) or a solid-phase extraction (SPE) method [96]. However, as mentioned before, the internal standard needs to correct for matrix effects as well, which is a problem separate from extraction recovery. If it has been proven that matrix effect is not observed, a structural analogue might well be appropriate. 

### 3.6. Metabolites

In some cases, quantification methods include drug metabolites as well, especially if metabolites have been known to be active and/or toxic. Midostaurin is biotransformed into active metabolites [113], and metabolites have been included in bioanalytical assays [66,67,68]. In these cases, one internal standard was used to correct for variation in analytical procedures for all analytes. As mentioned above, a recent method for midostaurin quantification was validated for high-throughput analysis for TDM, but none of the metabolites were quantified in this assay, whereas they were included in the older assays [69]. Some of these metabolites (CGP52421 and CGP62221) have been shown to be active, so it might have been interesting to include the metabolites in the assay [113]. Inclusion and quantification of metabolites in the assay might be valuable in metabolite profiling studies. However, this is only possible if the identities are already known, because reference standards should be available. When pre-clinical studies have demonstrated the formation of a particular major metabolite, it might be useful to include it in the validated assay. Additionally, an appropriate internal standard may be sought for the individual metabolites and parent compound. Often, the internal standard that is used to ensure accurate quantification of the parent drug is also used for the metabolite. Clearly, physiochemical properties are in this case not identical. For MS methods, matrix effects could be most effectively compensated for the quantification of the parent drug and its metabolites if the analytes co-elute with the SIL internal standard; however, the method should be sufficiently selective if the analytes are isomers, or if there is a risk that conjugates are re-converted into the parent drug in the source during ionization. 

## 4. Conclusions

Various analytical methods have been described to quantify a range of anticancer drugs of marine origin, including liquid chromatography with ultraviolet detection (LC-UV), coupled with fluorescence detection (LC-FLD), and in combination with mass spectrometry detection (LC-MS). A total of 35 compounds, of which 5 have been approved, were included in the current review. A total of 19 of those (excluding the ADCs and cytarabine) were finally entered into the PubMed database to search for quantification methods. These were found for eight of the compounds included. 

This review has selected many aspects of bioanalysis of the compounds isolated from sea-based organisms. The most used biomatrices include plasma and urine, and saliva, whole blood, feces and cells are occasionally analyzed as well. Sample cleanup is mostly done by PP, LLE, and SPE. The recent advances in this topic include the use of SLE, a method analogous to LLE. Samples are often concentrated during this step in the bioanalytical procedure. Some types of analysis might not be suitable for certain (groups of) drugs. For instance, we have seen that SPE has not been applied to the indocarbazoles and the marcrolides, and PP is most likely not useful for the extraction of depsipeptides from a biological sample. PP and LLE resulted in similar high recoveries of midostaurin, but only PP was applied to UCN-01 samples, also with good recoveries. The isoquinoline alkaloids seem to be extracted well from the matrices using LLE, SPE, and PP. 

Chromatographic separation is necessary to isolate the analyte of interest from endogenous interferences. Reversed-phase chromatography seems to be suitable for elution of all analytes discussed herein. C18 columns are clearly the most widespread columns used. Owing to most of the analytes’ hydrophobic properties, elution occurs at relatively high concentrations of organic solvents (≥50%), which is advantageous for the sensitivity of MS methods. 

Because this particular group of compounds has been the subject of clinical investigation for decades, the advances in bioanalysis are nicely demonstrated. We have seen a shift from LC-UV, a relatively cheap and widely available method, to LC-MS/MS, which has some advantages, most importantly, the superior sensitivity of LC-MS/MS compared with LC-UV. The sensitivity issues concerning the included compounds discussed in the present review include those arising from drugs administered at prolonged infusion times, which was the case for some of the depsipeptides, the isoquinolines, and one of the staurosporine derivatives, the existence of the two conformers that can occur for all proline-containing molecules, such as the depsipeptide plitidepsin, and the fact that the parent mass is not always the most pronounced ion observed in the mass spectrum, which has been described for the isoquinolines zalypsis, trabectedin, and lurbinectedin. Not only does the implementation of MS allow for the quantification of lower concentrations, but also it has the consequence that smaller sample volumes can be used for analysis. Although LLOQs as low as 1 ng/mL have been achieved using UV detection, sample volumes were quite large (500 μL). The superior sensitivity of LC-MS/MS compared with LC-UV also means that it is more suitable for metabolite quantification, because these are often present in circulation at even lower concentrations than the parent compound. Analytical run times are also reduced, due to the superior selectivity of LC-MS/MS. Internal standards are required to compensate for matrix effects resulting in improved accuracy and precision of the method, and the use of a SIL internal standard for MS bioanalysis is highly encouraged.

Continuous efforts are being made to discover, develop, and improve existing marine-derived drugs to treat cancer. Because ecological concerns are limiting new discoveries, more efforts are made in the laboratories, creating derivatives and analogues of the proven effective compounds discussed here. When these newly developed compounds show resemblance to the original compounds, the existing methods could be a basis for the newly validated assays. Depending on the drug properties, suitable cleanup, separation, and detection methods need to be developed and applied. The availability of resources and instrumentation is most likely the most common bottleneck: sometimes one can only work with what has been given.

Overall, this review provides information about the recent developments in bioanalysis of marine-derived anticancer compounds and can serve as a guide towards fast development and validation of new methods to quantify new marine-derived anticancer drugs.

## Figures and Tables

**Table 1:** Overview of marine-derived compounds approved or under investigation in clinical trials.

Name	Synonym (s)	Natural Product or Derivative	Origin	Administration	Dose	Indication	Status
**AGS-67E**		Derivative	Cyanobacterium *Caldora penicillata* [15]	intravenous infusion (i.v.)	Escalating doses	Lymphoid malignancies [18]	Phase I
**Brentuximab vedotin** (Adcetris^®^)	SGN-35/cAC10-vcMMAE	Derivative	Cyanobacterium *Caldora penicillata* [15]	i.v.	1.8 mg/kg [19]	Hodgkin lymphoma, Systemic anaplastic large cell lymphoma [19]	Approved
**Cytarabine** (Cytosar-U^®^/DepoCyte^®^)	Ara-C/Cytosine arabinoside/1-ß-D-Arabinofuranosylcytosine	Derivative	Sponge *Cryptotethya crypta* [12]	i.v./intrathecal	75–200 mg/m^2^ [20]	Acute myelogenous leukemia, Chronic myelogenous leukemia, Acute lymphoblastic leukemia non-Hodgkin’s lymphoma [20]	Approved
**Denintuzumab mafodotin**	SGN-CD19A/SGN-19A	Derivative	Cyanobacterium *Caldora penicillata* [15]	i.v.	3 mg/kg	Diffuse large B-cell lymphoma [21]	Phase II
**Depatuxizumab mafodotin**	ABT-414	Derivative	Cyanobacterium *Caldora penicillata* [15]	i.v.	1.25 mg/kg	Glioblastoma/Pediatric brain tumors [22]	Phase II
**Enfortumab vedotin**	ASG-22ME/ASG-22CE	Derivative	Cyanobacterium *Caldora penicillata* [15]	i.v.		Urothelial cancer [23]	Phase II
**Eribulin mesylate** (Halaven^®^)	E7389	Derivative	Sponge *Halichodria okadai* [12]	i.v.	1.23 mg/m² [24]	Breast cancer, Liposarcoma [24]	Approved
**Glembatumumab vedotin**	CDX-011/CR011-vcMMAE	Derivative	Cyanobacterium *Caldora penicillata* [15]	i.v.	1.9, 2.2 mg/kg	Breast cancer, Metastatic melanoma [25]	Phase II
**GSK2857916**	J6M0-mcMMAF	Derivative	Cyanobacterium *Caldora penicillata* [15]	i.v.	Escalating doses	Multiple myeloma [26]	Phase I
**Ladiratuzumab vedotin**	SGN-LIV1A	Derivative	Cyanobacterium *Caldora penicillata* [15]	i.v.	Escalating doses	Human epidermal growth factor receptor 2 (HER2)-negative breast cancer, triple negative breast cancer	Phase I
**Lurbinectedin** (Zepsyre^®^)	PM01183	Trabectedin analogue	Tunicate *Ecteinascidia turbinata* [12]	i.v.	3.2 mg/mL	Platinum-resistant ovarian cancer [27]	Phase III
**Marizomib**	Salinosporamide A/NPI-0052	Natural product	Marine actinomycete *Salinispora tropica* [12]	i.v.	0.5 mg/m^2^	Multiple myeloma, glioblastoma [28]	Phase II
**Midostaurin** (Rydapt^®^)	PKC412/CGP41251/N-benzoylstaurosporine	Staurosporine analogue	Bacterium *Streptomyces staurosporeus* [29]	oral	50 mg twice daily [30]	FLT3+ Acute myeloid leukemia [30]	Approved
**Pinatuzumab vedotin**	DCDT-2980S/RG7593	Derivative	Cyanobacterium *Caldora penicillata* [15]	i.v.	1.8, 2.4 mg/kg	Follicular lymphoma, Diffuse large B-cell lymphoma	Phase I
**Plinabulin**	NPI2358	Derivative	Marine fungus *Aspergillus* sp. [12]	i.v.	30 mg/m^2^	Non-small cell lung cancer [31]	Phase III
**Plitidepsin** (Aplidin^®^)	Dehydrodidemnin B	Natural product	Tunicate *Aplidium albicans* [12]	i.v.	5 mg/m^2^	Multiple Myeloma, Lymphoma [27]	Phase III
**PM060184**	PM0184/Plocabulin	Natural product	Sponge *Lithoplocamia lithistoides* [12]	i.v.	Starting dose 4 mg/m^2^	Breast cancer, Solid tumors [27]	Phase I
**Polatuzumab vedotin**	DCDS-4501A	Derivative	Cyanobacterium *Caldora penicillata* [15]	i.v.	1.8, 2.4 mg/kg	Non-Hodgkin’s lymphoma, B-cell lymphoma [32]	Phase II
**Tisotumab vedotin**	HuMax-TF-ADC/HuMab-TF-011-vcMMAE/TF-011-vcMMAE	Derivative	Cyanobacterium *Caldora penicillata* [15]	i.v.	Escalating doses	Solid tumors [33]	Phase I
**Trabectedin** (Yondelis^®^)	ET-743	Natural product	Tunicate *Ecteinascidia turbinate* [12]	i.v.	1.1/1.5 mg/m^2^ [34]	Soft tissue sarcoma [34]	Approved

**Table 2:** Marine-derived anticancer drugs for which trials were discontinued or for which no active trials (NAT) were found in the ClinicalTrials.gov database.

Name	Synonym (s)	Natural Product or Derivative	Origin	Discontinued/Inactive	Reason for Discontinuation
**ASG-15ME**	AGS15E	Derivative	Cyanobacterium *Caldora penicillata* [15]	Discontinued	Unspecified [35]
**Becatecarin**	XL-119/NSC 655649/BMY 27557/BMS 181176	Rebeccamycin analogue	Marine actinomycete *Saccharothrix aerocolonigenes* [36]	Discontinued	Not superior to existing therapies [37]
**CEP-2563**	KT-8391	Staurosporine derivative		NAT	
**Didemnin B**		Natural product	Tunicate *Trididemnin cyanophorum* [38]	Discontinued	Toxicity [39]
**Edotecarin**	J-107088/PF-804950/PHA-782615/ED-749	Derivative		NAT	
**Elisidepsin** (Irvalec^®^)	PM02734	Structural analogue	Mollusk *Elysia rufescens* [40]	Discontinued	Strategic [41]
**Enzastaurin**	LY317615	Staurosporine derivative	Bacterium *Streptomyces staurosporeus*	Discontinued	Lack of efficacy [42]
**Indusatumab vedotin**	MLN-0264/TAK-0264	Derivative	Cyanobacterium *Caldora penicillata* [15]	Discontinued	Lack of efficacy [43]
**Lestaurtinib**	CEP-701	Staurosporine derivative	Bacterium *Streptomyces staurosporeus*	Discontinued	Strategic [41]
**Lifastuzumab vedotin**	DNIB0600A/Anti-NaPi2B ADC/RG7599	Derivative	Cyanobacterium *Caldora penicillata* [15]	Discontinued	Lack of efficacy [44]
**Pipecolidepsin A**		Natural product	Sponge *Homophymia lamellose* [13]	NAT	
**Stellatolide A**		Natural product	Sponge *Ecionemia acervus* [45]	NAT	
**UCN-01**	7-hydroxystaurosporine	Staurosporine analogue	Bacterium *Streptomyces staurosporeus* [29]	NAT	
**Vandortuzumab vedotin**	DSTP-3086 S/RG-7450/thio-antiSTEAP1-MC-vc-PAB-MMAE	Derivative	Cyanobacterium *Caldora penicillata* [15]	NAT	
**Zalypsis**	PM00104/PM-10450	Derivative	Sponge *Netropsia* sp. [15]	NAT	

**Table 3:** Overview of published bioanalytical methods to quantify marine-derived anticancer drugs in biological matrices.

Compound	Matrix	Sample Pre-Treatment	Stationary Phase	Mobile Phase	Detection	Internal Standard	Linear Range	LOD	Metabolites	Ref.
Elisidepsin	Plasma (Dog)	LLE (ethyl acetate)	YMC Pro C18, S-5 (50 × 2.0 mm, 120 Å)	A: 5 mM ammonium acetate, 0.1% FA in H_2_O B: 0.1% FA in MeOH	MS/MS	^2^H_8_-PM02734 (SIL)	0.05–100 ng/mL		No	[62]
Eribulin mesylate	Plasma Urine (Human)	LLE (ethyl acetate/MeOH/EtOH)	Polaris^®^ C18 (30 × 2.0 mm, 3 μm)	A: 0.1% FA in H_2_O-ACN (87:13, *v*/*v*) B: 0.1% FA in THF-ACN (30:70, *v*/*v*)	MS/MS	ER-076349 (Structural analogue)	0.2–100 ng/mL		No	[63]
Eribulin mesylate	Plasma Whole blood Urine Feces (Human)	P/WB/U: LLE (ethyl acetate/MeOH/EtOH) F: dilution (ACN)	Polaris^®^ C18-A (30 × 2.0 mm, 3 μm)	A: 0.1% FA in H_2_O B: 0.1% FA in ACN	MS/MS	ER-076349 (Structural analogue)	0.2–100 ng/mL (P); 0.5–100 ng/mL (WB/U); 100–25,000 ng/mL (F)		No	[64]
Lurbinectedin	Plasma (Cynomolgus monkey Dog Mice Mini-pig Rat)	SLE (TBME)	ACE C18 PFP (30 × 2.1 mm, 3 μm)	A: 0.1% FA in H_2_O B: 0.1% FA in ACN	MS/MS	PM040038 (SIL)	0.1–100 ng/mL	0.025 ng/mL	No	[65]
Midostaurin	Plasma (Human)	LLE (diisopropyl ether)	μBondapak RP-18 (300 × 3.9 mm, 10 μm)	ACN-0.001 M ammonium acetate in H_2_O, pH 4.0 (45:55, *v*/*v*)	FLD 286/386 nm	CGP 41 126 (Structural analogue)	1–1000 ng/mL	0.5 ng/mL	CGP 50 723; CGP 50 750; CGP 52 421	[66]
Midostaurin	Plasma (Human)	LLE (diisopropyl ether)	RP LiChrospher C18 end-capped (125 × 4.0 mm, 5 μm)	A: ACN B: 445 μL TEA in 1 L phosphate buffer, pH 3.6	FLD 286/386 nm	CGP 41 126 (Structural analogue)	0.2–1000 ng/mL	0.1 ng/mL	CGP 50 673; CGP 50 723; CGP 50 750; CGP 52 421	[67]
Midostaurin	Plasma (Human)	LLE (diethyl ether)	Prodigy ODS-2 (150 × 3.2 mm, 5 μm)	0.4 mL Titriplex III-solution in MeOH-ACN-0.05 M ammonium acetate in water (40:26:34, *v*/*v*/*v*)	FLD 286/386 nm	N-phenyl-1- naphthylamine	10–10,000 ng/mL	10 ng/mL	CGP 52421e1; CGP 52421e2; CGP 62221; CGP 62221e1; CGP 62221e2;	[68]
Midostaurin	Plasma (Human)	PP (MeOH)	SunFire bonded and end-capped C18 silica (150 × 2.1 mm, 3.5 μm)	A: 10 mM ammonium formate, 0.1% FA in H_2_O B: 0.1% FA in ACN	MS/MS	Midostaurin-d5 (SIL)	75–2500 ng/mL		No	[69]
Plitidepsin	Plasma Urine (Rat)	PP (0.1% FA in ACN) + LLE (chloroform)	RP C18 Hypersil-5 ODS (100 × 3.0 mm; 5 μm, 120 Å)	A: 0.5% FA in H_2_O B: 0.5% FA in ACN	MS/MS	Didemnin B (Structural analogue)	5–100 ng/mL (P); 1.25–125 ng/mL (U)	1 ng/mL (P); 0.5 ng/mL (U)	No	[70]
Plitidepsin	Plasma (Mice)	Derivatization + SPE	Symmetry C18 (100 × 4.6 mm, 3.5 μm)	ACN–water–TFA (47:52.9:0.1, *v*/*v*/v)	FLD 410/560 nm	None	2–100 ng/mL		No	[71]
Plitidepsin	Plasma (Human)	LLE (TBME)	Zorbax Bonus-RP (50 × 2.0 mm, 5 μm)	0.1% FA in ACN-5 mM ammonium acetate, 0.1% FA in H_2_O (80:20, *v*/*v*)	MS/MS	Didemnin B (Structural analogue)	0.05–50 ng/mL		No	[72]
Plitidepsin	Plasma Whole Blood Urine (Human)	PP (0.1% FA in ACN) + LLE (chloroform)	Hypersil-5 ODS (100 x 3.0 mm, 5 μm, 120 Å)	A: 0.5% FA in ACN B: 0.5% FA in H_2_O	MS/MS	Didemnin B (Structural analogue)	1–250 ng/mL	0.25 ng/ml	No	[73]
Plitidepsin	Whole Blood (Human)	Derivatization + SPE	Symmetry C18 (100 × 4.6 mm, 3.5 μm)	ACN-0.1% TFA in H_2_O (50:50, *v*/*v*)	FLD 410/560 nm	None	2–100 ng/mL		No	[74]
Plitidepsin	Plasma Whole Blood Urine (Human)	LLE (TBME)	SunFire C18 (50 × 2.1 mm, 5 μm)	A: 5 mM ammonium acetate, 0.1% FA in H_2_O B: 0.1% FA in ACN	MS/MS	(PM130461) ^13^C_5_-^15^N-plitidepsin (SIL)	0.1–100 ng/mL		No	[75]
Trabectedin	Plasma (Human)	SPE	Zorbax SB-C18 column (75 × 4.6 mm, 3.5 μm)	ACN–25 mM phosphate buffer, pH 5.0 (70:30, *v*/*v*)	UV 210 nm	POB	1–50 ng/mL		No	[76]
Trabectedin	Plasma (Human)	SPE	Zorbax Rx-C18 (150 × 2.1 mm, 5 μm)	MeOH-5 mM ammonium acetate, 0.4% FA in H_2_O (75:25, *v*/*v*)	MS/MS	ET-729 (Structural analogue)	0.01–2.5 ng/mL		No	[77]
Trabectedin	Plasma (Human)	PP (MeOH)	Zorbax Rx-C18 (150 × 2.1 mm, 5 μm)	MeOH–H_2_O (85:15, *v*/*v*)	MS/MS	^2^H_3_-ET-743 (SIL)	0.05–2.5 ng/mL		No	[78]
Trabectedin	Plasma (Human)	PP (HCl in MeOH)	Accucore XL C18 (50 × 2.1 mm, 4 μm)	A: 10 mM ammonium acetate in H_2_O, pH 6.8 B: MeOH	MS/MS	^2^H_3_-ET-743 (SIL)	0.025–1.0 ng/ml		No	[79]
Trabectedin	Liver cells Tumor cells (Mice)	Lysis	Accucore XL C18 (50 × 2.1 mm, 4 μm)	A: 10 mM ammonium acetate in H_2_O, pH 6.8B: MeOH	MS/MS	^2^H_3_-ET-743 (SIL)	0.1–3 ng/mL (TC); 0.25–6 ng/mL (LC)		No	[80]
UCN-01	Plasma Urine (Human)	PP (ACN)	AM-312 ODS (150 × 6.0 mm S-5mm, 120 Å)	ACN–0.1% TEA in 0.05 M phosphate buffer, pH 7.3 (50:50, *v*/*v*).	FLD 310/410 nm	Staurosporine (Structural analogue)	0.2–100 ng/mL (P); 1–400 ng/mL (U)		No	[81]
UCN-01	Plasma Saliva (Human)	PP (ACN)	Nova-Pak Phenyl (150 × 3.9 mm, 4 μm)	A: 0.05 M ammonium acetate in H_2_O, pH 4.15 B: ACN	UV (P) 295 nm FLD (Sal) 290/400 nm	Umbelliferone	200–20,000 ng/mL (P); 4–200 ng/mL (S)		No	[82]
UCN-01	Plasma (Human)	PP (ACN)	Nova-Pak Phenyl (150 × 3.9 mm, 4 μm, 60 Å)	ACN–0.5 M ammonium acetate, 0.2% TEA in H_2_O (45:55, *v*/*v*)	FLD 310/410 nm	Staurosporine (Structural analogue)	200–30,000 ng/mL	0.1 μg/mL	No	[83]
Zalypsis	Plasma (Dog Human Mice Rat)	LLE (TBME)	Zorbax SB-C18 (50 × 2.1 mm, 5 μm, 80 Å)	A: 5 mM ammonium acetate, 0.1% FA in H_2_O B: 0.1% FA in MeOH	MS/MS	^13^C_2_,^2^H_3_-PM00104 (SIL)	0.01–5 ng/mL		No	[84]

ACN: acetonitrile; EtOH: ethanol; F: Feces; FA: formic acid; FLD: fluorescence detection with excitation and emission wavelengths; HCl: hydrochloric acid; H_2_O: water; LC: liver cells; LLE: liquid-liquid extraction; LOD: limit of detection; MeOH: methanol; MS/MS: tandem mass spectrometry; P: plasma; PFP: pentafluorophenyl; POB: propyl-p-hydroxybenzoate; PP: protein precipitation; RP: reversed phase; S: saliva; SIL: stable isotopically labeled; SLE: supported liquid extraction; SPE: solid phase extraction; TBME: *tert*-butyl methyl ether; TC: tumor cells; TEA: triethylamine; TFA: trifluoroacetic acid; THF: tetrahydrofuran; U: urine; UV: ultraviolet detection with detector wavelength; and WB: whole blood.
